# Blue Pseudochromhidrosis Secondary to Cellulitis Caused by Pseudomonas stutzeri

**DOI:** 10.7759/cureus.105654

**Published:** 2026-03-22

**Authors:** Nathalia Madureira Ferraz, Danielli Oliveira da Costa Lino

**Affiliations:** 1 Department of Internal Medicine, Hospital de Messejana Dr. Carlos Alberto Studart Gomes, Fortaleza, BRA; 2 Department of Internal Medicine, Faculdade Estácio de Quixadá, Instituto de Educação Médica (IDOMED), Quixadá, BRA; 3 Department of Cardiology, Hospital de Messejana Dr. Carlos Alberto Studart Gomes, Fortaleza, BRA

**Keywords:** blue sweat, chromhidrosis, chromogenic bacteria, colored sweat, pseudochromhidrosis, pseudomonas stutzeri

## Abstract

Pseudochromhidrosis is an uncommon dermatological condition in which eccrine sweat, normally colorless, acquires pigmentation upon contact with exogenous chromogenic substances on the skin surface, including chromogenic microorganisms. We report the case of an 88-year-old man who presented with bluish sweating and blue staining of the skin and nails seven days after sustaining trauma to his right leg at a construction site, which progressed to cellulitis with an ulcerated lesion. Alcohol cleansing temporarily removed the discoloration, which reappeared shortly thereafter. Microbiological culture of the ulcerated lesion revealed *Pseudomonas stutzeri* susceptible to quinolones. Targeted therapy with intravenous levofloxacin was initiated, resulting in the marked improvement of the bluish discoloration within 24 hours. A review of the available literature did not identify prior reports of *Pseudomonas stutzeri* associated with infectious pseudochromhidrosis. This case expands the recognized infectious spectrum of this condition and highlights the importance of microbiological evaluation in patients presenting with anomalous sweat discoloration.

## Introduction

Pseudochromhidrosis is an uncommon dermatological condition characterized by the discoloration of initially colorless eccrine sweat following contact with exogenous chromogenic substances on the skin surface, including pigment-producing microorganisms [1,2]. Chromhidrosis is traditionally classified into apocrine, eccrine, and pseudochromhidrosis, each with distinct pathophysiologic mechanisms and clinical implications [1,3].

In apocrine chromhidrosis, sweat is intrinsically pigmented due to lipofuscin accumulation within apocrine gland secretory cells [1,2]. In contrast, eccrine chromhidrosis results from the excretion of water-soluble pigments, medications, or dyes through the eccrine glands [1-3]. Pseudochromhidrosis differs in that sweat becomes discolored only after secretion, as a result of interaction with external chromogenic agents [1,2].

Infectious pseudochromhidrosis represents a recognized subtype and results from the colonization of the skin by pigment-producing microorganisms capable of interacting with eccrine sweat [2,4]. Reported causative organisms include *Bacillus* species, *Corynebacterium* species, *Serratia marcescens*, and *Pseudomonas aeruginosa *[2]. Recent case reports have described infectious pseudochromhidrosis in association with alterations in the cutaneous microbiota [4].

*Pseudomonas stutzeri *is a Gram-negative, aerobic, non-fermenting bacillus widely distributed in soil and water. Although historically considered of low virulence, it has increasingly been recognized as an opportunistic pathogen in individuals with predisposing factors such as underlying disease, prior surgery, trauma, or immunocompromising conditions [5-7]. However, it has not been previously reported in association with infectious pseudochromhidrosis. We describe a case of blue pseudochromhidrosis occurring secondary to cellulitis in which *Pseudomonas stutzeri* was isolated from the affected site, thereby expanding the spectrum of infectious etiologies linked to this condition.

## Case presentation

An 88-year-old man presented to the emergency department seven days after sustaining trauma to the posterior aspect of his right leg at a construction site. At presentation, he reported bluish sweating that began after the injury. The affected area subsequently evolved into clinically evident cellulitis of the right lower limb. He denied previous similar episodes and reported no recent ingestion of unusual foods or beverages, exposure to dyes or chemicals, or initiation of new medications. The patient had a medical history of hypertension and prediabetes, with no other known comorbidities.

**Figure 1:**
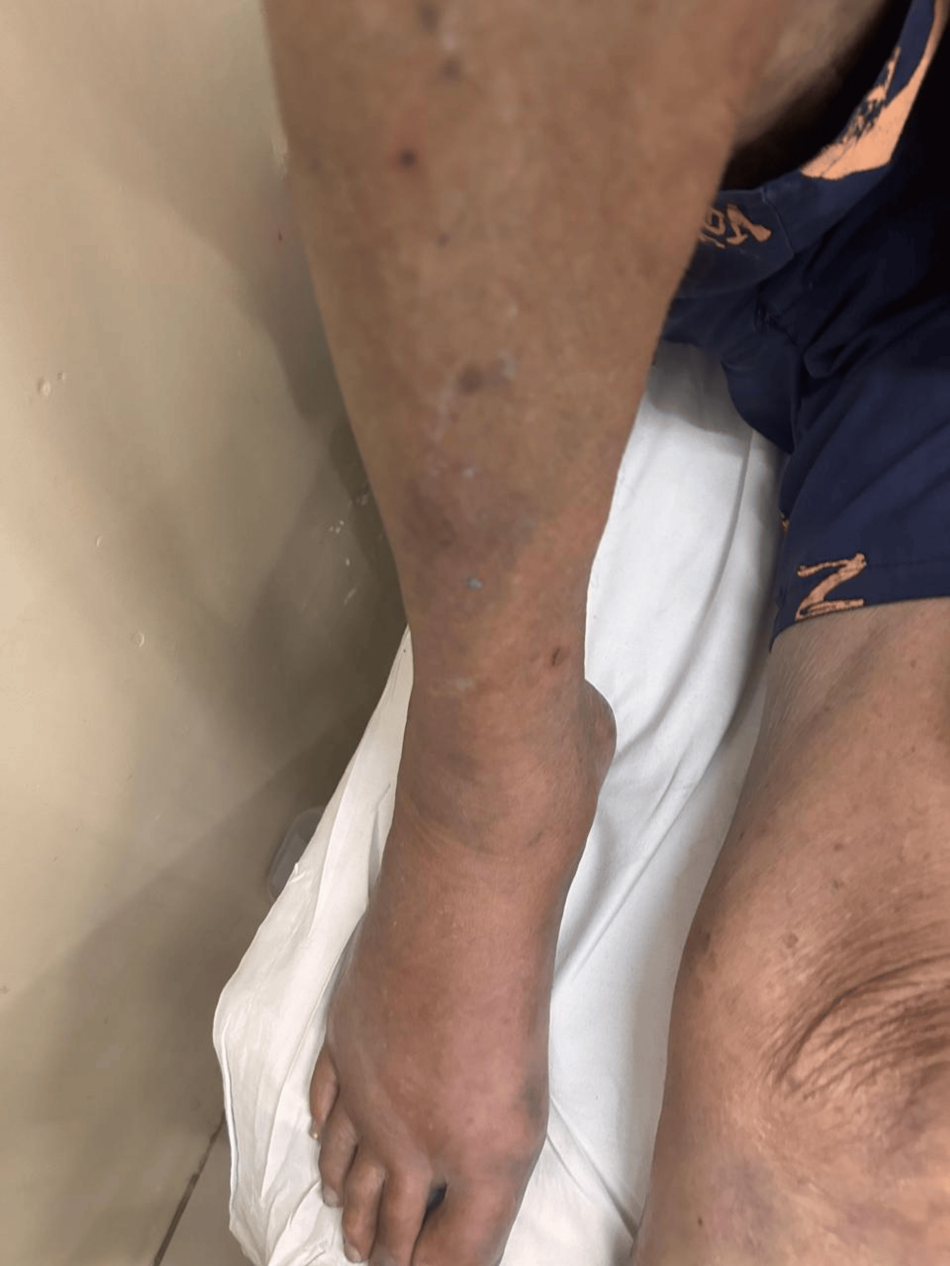
Blue cutaneous discoloration of the right lower limb

**Figure 2:**
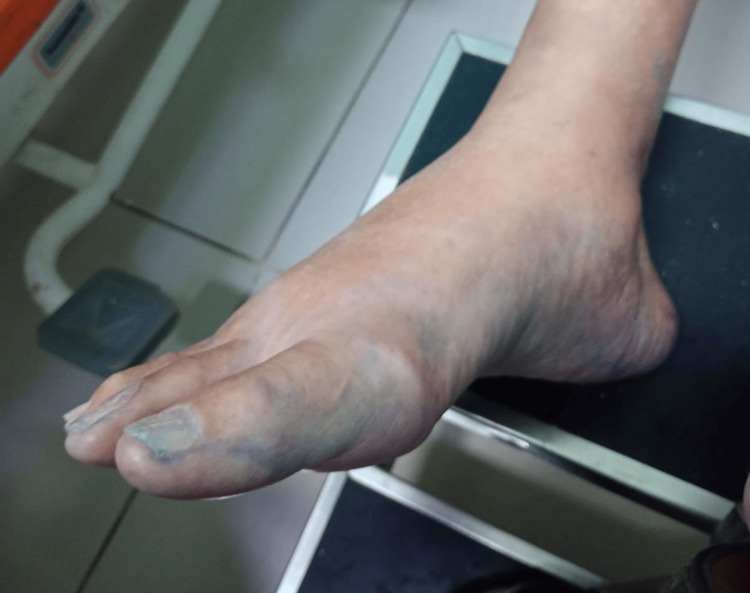
Blue cutaneous discoloration of the right foot at presentation

**Figure 3:**
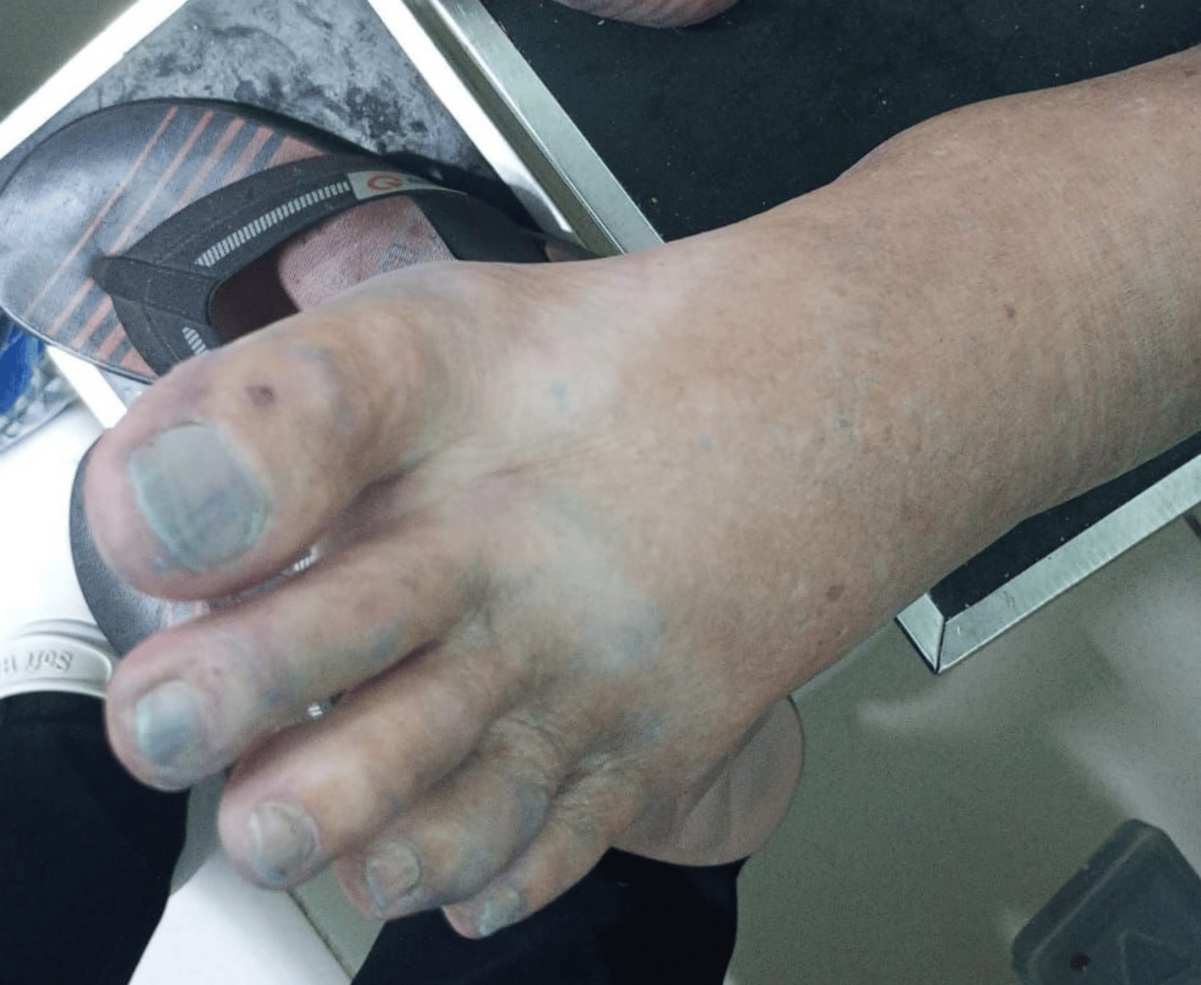
Blue discoloration of the toes and toenails of the left foot at presentation

**Figure 4:**
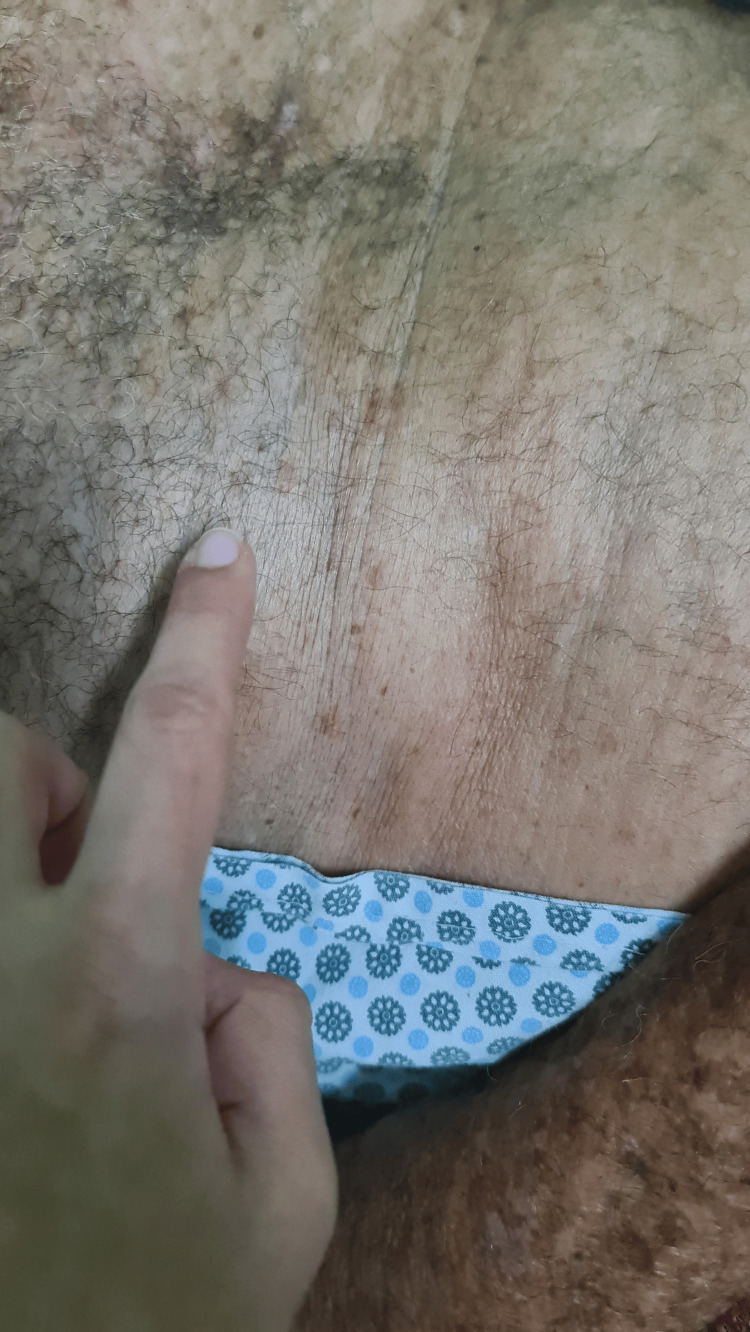
Blue cutaneous discoloration of the upper abdominal quadrants

**Figure 5:**
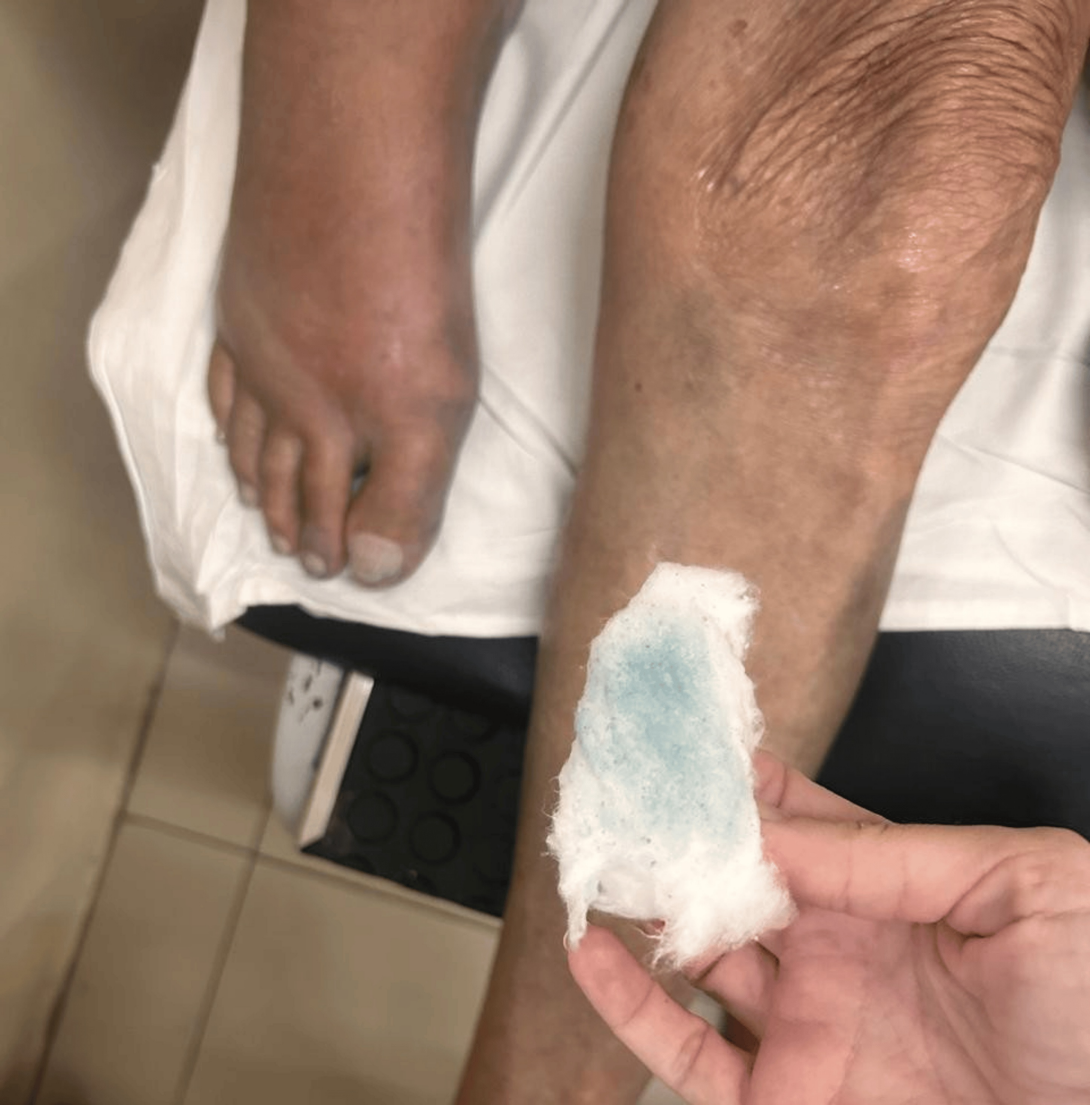
Transfer of blue pigment to cotton following alcohol cleansing

On physical examination, he was afebrile and hemodynamically stable. Blue staining was observed on the lower limbs (Figure 1), feet (Figure 2), nails (Figure 3), abdomen (Figure 4), and hands. An ulcerated lesion with surrounding erythema and edema was noted on the posterior aspect of the right leg. The pigmentation was removable with alcohol cleansing and transferred to cotton (Figure 5), reappearing after perspiration.

**Figure 6:**
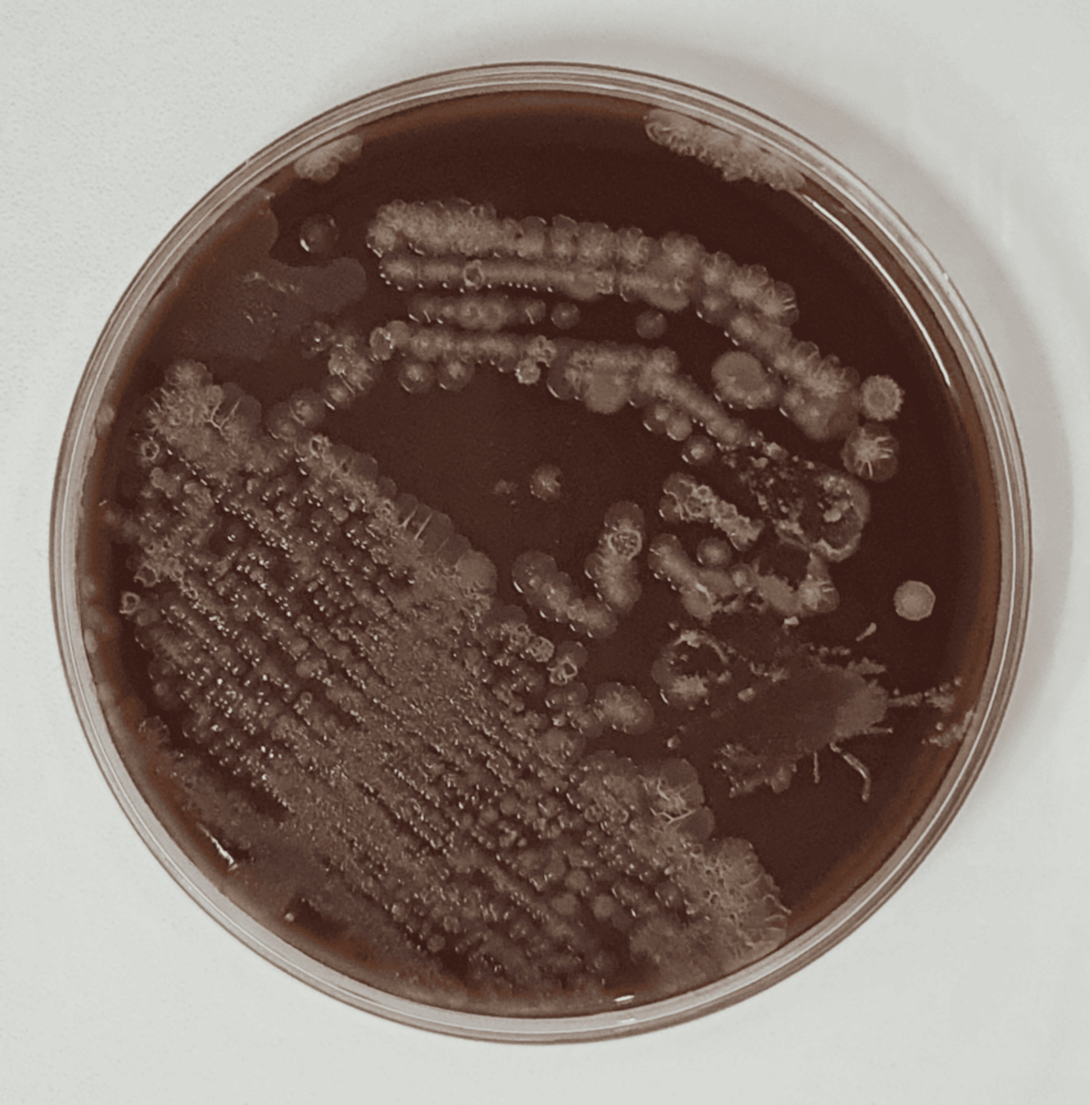
Growth of Pseudomonas stutzeri on chocolate agar *Pseudomonas stutzeri* isolated from the ulcerated lesion, grown on chocolate agar, showing irregular, dry, and wrinkled colonies consistent with its characteristic morphology.

Initial laboratory tests revealed mild anemia, leukocytosis, and elevated C-reactive protein levels (Table 1). Blood and urine cultures were negative. Culture obtained from the ulcerated lesion yielded *Pseudomonas stutzeri* (Figure 6), which was susceptible to quinolones.

**Table 1:** Key laboratory findings on admission

Parameter	Admission value	Reference range
Hemoglobin (g/dL)	11.9	13.0-18.0
White blood cell count (/mm³)	13.5	4,000-10,000
Platelet count (/mm³)	260	140,000-500,000
C-reactive protein (mg/dL)	13.8	<0.5

**Figure 7:**
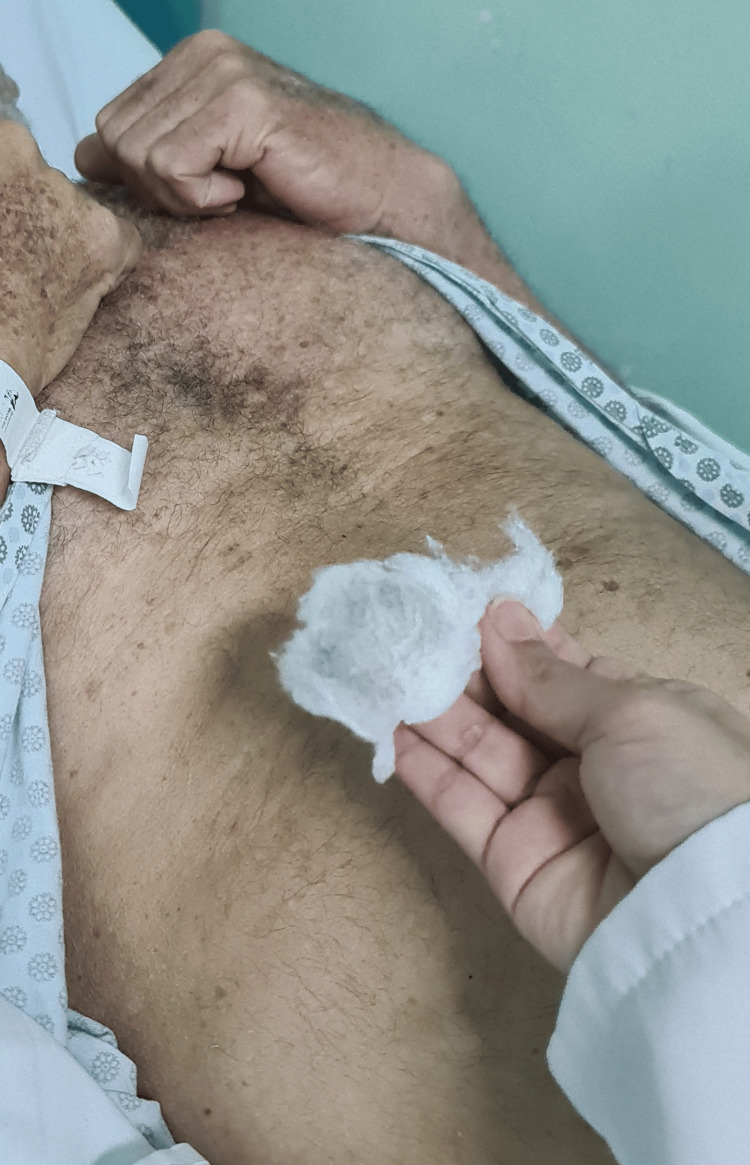
Cotton after alcohol cleansing 24 hours after the initiation of antibiotic therapy

**Figure 8:**
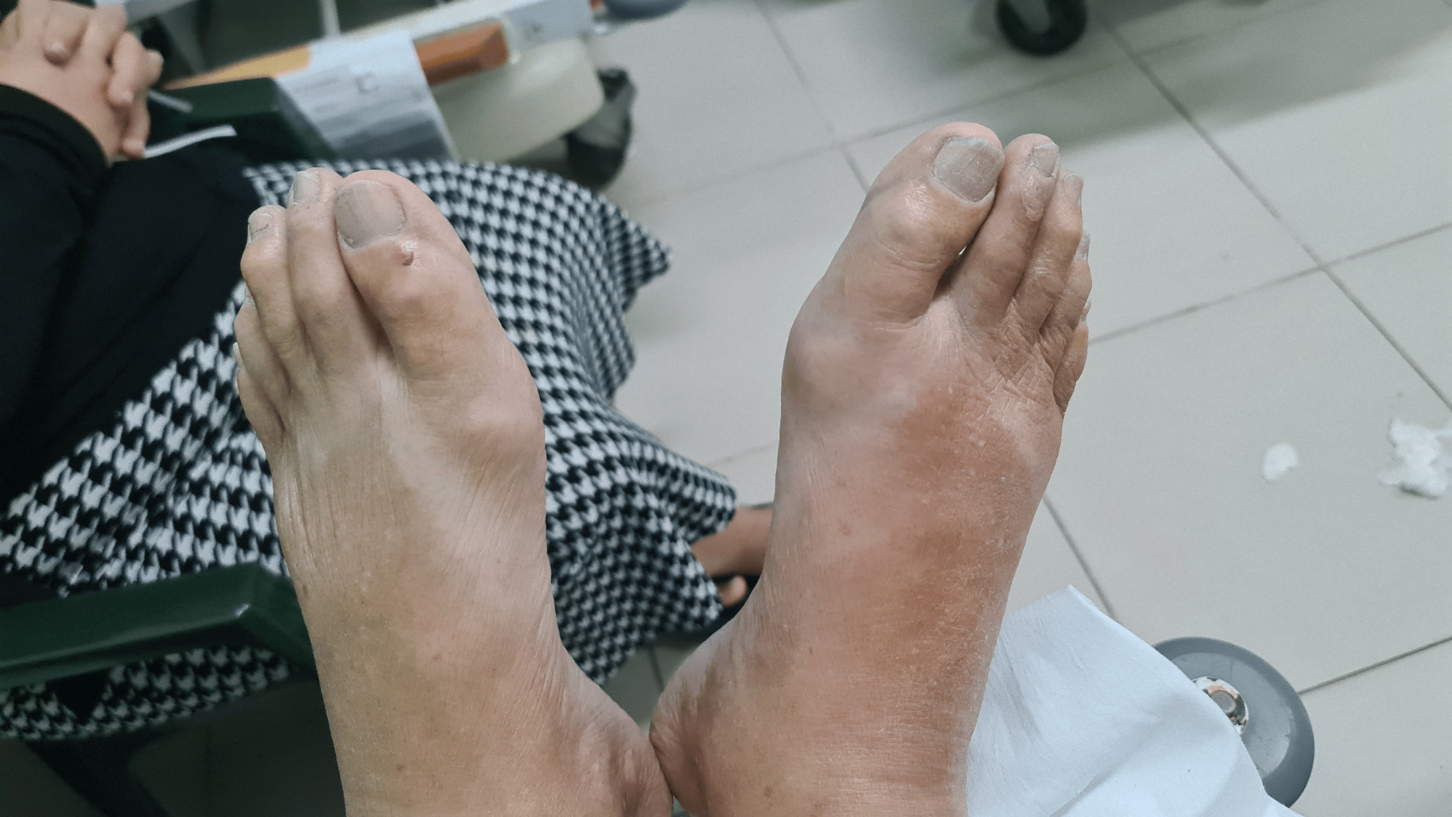
Feet and toenails 24 hours after antibiotic therapy

Intravenous levofloxacin was initiated. A marked reduction in blue discoloration was observed within 24 hours of antibiotic initiation (Figures 7-8). Subsequent laboratory tests demonstrated normalization of the leukocyte count and a progressive decline in inflammatory markers, particularly C-reactive protein (Table 2). Doppler ultrasonography of the right lower limb showed no vascular abnormalities. The cellulitis improved progressively during hospitalization. The patient was discharged after seven days of inpatient treatment with oral levofloxacin to complete a 10-day course of antibiotic therapy.

**Table 2:** Follow-up laboratory findings after antibiotic therapy

Parameter	Admission value	Reference range
Hemoglobin (g/dL)	10.0	13.0-18.0
White blood cell count (/mm³)	7.56	4,000-10,000
Platelet count (/mm³)	255	140,000-500,000
C-reactive protein (mg/dL)	5	<0.5

## Discussion

The clinical presentation of blue cutaneous discoloration in this patient required careful differentiation among the recognized causes of colored sweat. The generalized distribution of discoloration, including areas not rich in apocrine glands, was less consistent with apocrine chromhidrosis, which typically affects the axillae and face [1]. Eccrine chromhidrosis secondary to ingestion of medications, dyes, or exogenous substances was also considered; however, the patient reported no recent changes in medication use or ingestion of substances outside his usual dietary or pharmacologic habits. The temporal relationship between traumatic skin disruption, subsequent cellulitis, and the onset of blue discoloration suggested an infectious mechanism.

Infectious pseudochromhidrosis has been described in association with pigment-producing bacteria capable of altering the appearance of eccrine sweat [2,3]. In this case, *Pseudomonas stutzeri *was isolated from an ulcerated cellulitic lesion temporally associated with the onset of discoloration. The rapid reduction in pigmentation observed within 24 hours of initiating targeted antimicrobial therapy, together with the normalization of inflammatory markers, supports a clinically meaningful association.

Although traditionally regarded as an organism of low virulence, *Pseudomonas stutzeri *has been increasingly recognized as an opportunistic pathogen in individuals with predisposing factors such as underlying disease, prior surgery, trauma, or immunocompromising conditions [5-7]. In the present case, traumatic disruption of the skin barrier likely facilitated localized bacterial proliferation, enabling the interaction between microbial metabolites and eccrine sweat.

A review of the available literature did not identify previously documented cases of infectious pseudochromhidrosis associated with *Pseudomonas stutzeri*. This finding expands the recognized infectious spectrum of the condition and underscores the importance of microbiological evaluation in patients presenting with unexplained colored sweat.

While the clinical course and therapeutic response suggest a causative association, the precise biochemical mechanisms linking *Pseudomonas stutzeri *to the chromogenic phenomenon remain to be clarified. Future studies may help elucidate the underlying pathophysiology.

## Conclusions

This case demonstrates that infectious pseudochromhidrosis may occur in association with *Pseudomonas stutzeri *isolated from a localized cellulitic lesion. The temporal relationship between infection and discoloration, together with prompt clinical and laboratory improvement following targeted antimicrobial therapy, supports a clinically meaningful association. Detailed microbiological investigation and early directed treatment are essential for accurate diagnosis and effective management. This report contributes to the expanding spectrum of bacterial isolates associated with anomalous sweat discoloration.
